# Immune Modulation as a Key Mechanism for the Protective Effects of Remote Ischemic Conditioning After Stroke

**DOI:** 10.3389/fneur.2021.746486

**Published:** 2021-12-09

**Authors:** Sima Abbasi-Habashi, Glen C. Jickling, Ian R. Winship

**Affiliations:** ^1^Neuroscience and Mental Health Institute, University of Alberta, Edmonton, AB, Canada; ^2^Division of Neurology, Faculty of Medicine, University of Alberta, Edmonton, AB, Canada; ^3^Neurochemical Research Unit, Department of Psychiatry, University of Alberta, Edmonton, AB, Canada

**Keywords:** cerebral ischemia, collateral circulation, remote ischemic conditioning (RIC), inflammatory response, extracellular vesicles (EVs), microRNAs

## Abstract

Remote ischemic conditioning (RIC), which involves a series of short cycles of ischemia in an organ remote to the brain (typically the limbs), has been shown to protect the ischemic penumbra after stroke and reduce ischemia/reperfusion (IR) injury. Although the exact mechanism by which this protective signal is transferred from the remote site to the brain remains unclear, preclinical studies suggest that the mechanisms of RIC involve a combination of circulating humoral factors and neuronal signals. An improved understanding of these mechanisms will facilitate translation to more effective treatment strategies in clinical settings. In this review, we will discuss potential protective mechanisms in the brain and cerebral vasculature associated with RIC. We will discuss a putative role of the immune system and circulating mediators of inflammation in these protective processes, including the expression of pro-and anti-inflammatory genes in peripheral immune cells that may influence the outcome. We will also review the potential role of extracellular vesicles (EVs), biological vectors capable of delivering cell-specific cargo such as proteins and miRNAs to cells, in modulating the protective effects of RIC in the brain and vasculature.

## Introduction

The incidence, mortality, and prevalence of neurological disorders are increasing worldwide, primarily because of the growing elderly population (1). Stroke is one of the most common neurovascular conditions with a prevalence of 101.5 million people worldwide (2021 Heart Disease and Stroke Statistical Update) (1). Of these strokes, 76% were classified as ischemic stroke (~77.2 million), ~ 20% as intracerebral hemorrhage (~20.7 million), and about 8% as subarachnoid hemorrhage (8.4 million) (1). Acute ischemic stroke (AIS) occurs when a major artery that supplies oxygen and nutrients to the brain becomes obstructed, leading to the formation of two injury zones: The ischemic core and the “penumbra.” The infarct core is severely hypoperfused, such that neurons undergo rapid and irreversible necrotic cell death (2). In response to ischemia and cell death in the core, inflammatory signals are released into the peripheral circulation, attracting immune cells to the damaged area and exacerbating the inflammatory response. The core of the ischemic region is surrounded by a relatively hypoperfused zone called the penumbra, which defines the tissue at risk for further infarction (3). Because cell death in this penumbral region occurs gradually, it is possible to rescue this peri-infarct area in the hyper-acute phase of AIS prior to cell death and infarct expansion.

One important factor contributing to the viability of penumbral tissue is the existence of strong collateral circulation that reduces ischemia in the penumbra, reducing injury, and improving the clinical outcomes (4). Pial collateral vessels—also called leptomeningeal collaterals–are auxiliary vascular networks on the brain surface that connect the distal part of major branches of the anterior and posterior cerebral artery (ACA, PCA) with the distal branches of the middle cerebral artery (MCA). These vascular anastomoses provide oxygen and essential nutrients *via* retrograde blood flow to the deprived ischemic tissue when the primary artery is blocked (5). Currently approved treatments for AIS, such as thrombolysis through recombinant tissue plasminogen activator (rtPA) administration or recanalization *via* mechanical endovascular treatment (EVT, i.e., mechanical thrombectomy), work in a time dependent manner and have a limited therapeutic window (6). While good collateral blood flow can extend this window, rapid restoration of flow to the brain remains the best treatment for acute stroke. However, this is restricted to ~10–20 percent of stroke sufferers who can make it to a primary stroke treatment center in time. Treatments that can improve collateral blood flow may extend the window for recanalization therapy and improve outcome for stroke patients (5).

Even after flow is restored in an occluded cerebral vessel, cellular injury can be exacerbated by reperfusion injury (7). Recanalization of the occluded artery can lead to damage to the integrity of the capillary endothelium, known as ischemia/reperfusion (IR) injury. Restored flow can increase BBB permeability when a high blood volume re-enters the already collapsed vasculature. Following this reperfusion, activated endothelial cells (ECs) produce reactive oxygen species (ROS), which further triggers the influx of inflammatory cells to the ischemic site (8). Increased leukocyte stimulation, trafficking and release of proinflammatory chemoattractant substances amplifies local inflammation. Elevated expression of adhesion molecules on ECs can further potentiate interactions between circulating blood cells and ECs, particularly neutrophil-endothelial interactions that can lead to neutrophil aggregation in the capillary bed (9). Reducing IR injury is key to improving outcome after recanalization therapy. So far, several approaches have been attempted to inhibit leukocytes aggregation and attenuate IR injury, but none have proven effective in clinic (10). Additional therapies are urgently needed to protect brain tissue from the ischemic and post-reperfusion damage. One such approach may be remote ischemic conditioning (RIC) (11, 12). RIC has shown to be a clinically safe and straightforward intervention which helps to attenuate the detrimental effects of ischemia. Multiple molecular signaling pathways contribute to the protective effects of RIC against reperfusion injury, with key signaling pathways converging on transcription factors that regulate cell survival and apoptosis (13). Of these signaling cascades, the reperfusion injury salvage kinase (RISK) and the survivor activating factor enhancement (SAFE) pathways are well-characterized. Below, we will review RIC for stroke treatment, its established mechanisms, and discuss RIC induced modulation of inflammatory immune cells and their gene expression profiles.

## RIC: Concept and Origin

Remote Ischemic Conditioning (RIC) is a therapy that involves brief, intermittent episodes of sublethal ischemia and reperfusion that is applied to a peripheral tissue, organ or a vascular territory. This peripheral signal is then transmitted to the distal target organ (e.g., brain or heart) to relay protection against prolonged ischemia and subsequent IR injury (14, 15).

In 1986, ischemic preconditioning was described by Murry et al. in relation to cardiac ischemia (16). A preconditioning (PC) intervention was directly applied to the dog heart *via* four cycles (each for 5 min) of alternative occlusion/reflow of the left anterior descending (LAD) coronary artery prior to initiation of 40 min cardiac ischemia (16). Their results showed that PC was associated with a considerable reduction in myocardial infarction size. However, in another animal cohort, the same PC protocol preceding 3 h of sustained coronary occlusion failed to salvage the heart tissue injury, suggesting that PC has a protective time window and it may only delay the cellular death up to a few hours and then dissipates (16). Thereafter, additional investigations advanced the theory of “two time windows for protection” based upon these results (17–19). The early phase of protection occurs immediately, within minutes after the PC application, and lasts for ~3 h. It is thought that the early phase is mainly caused by rapid alterations in protein kinase signaling pathways that converge on the mitochondria to stop the apoptotic pathways (20, 21). The late phase starts 18–24 h after PC and lasts for ~4 days. The protection during the late period is probably due to *de novo* synthesis of proteins that are involved in inflammation, ischemia and vascular dynamics (12, 22, 23), and the suppression of genes involved in IR injury.

In 1993 the conditioning concept was extended to remote ischemic conditioning (RIC), in which ischemia is induced to an organ far from the target organ, often using a blood pressure cuff, offering a safe and feasible approach (24).

## RIC Modalities

The remote application of RIC provides a safe, non-invasive and clinically applicable method, often involving intermittent cycles of inflation and deflation of a blood pressure cuff around the upper arm in humans and upper hind limb in preclinical studies in rodents (25). RIC has been used in three temporal windows during or after cerebral ischemia: (1) remote ischemic preconditioning (RIPreC) is applied prior to the injurious ischemia. While less practical as a therapeutic approach, because the stroke event is not always predictable, RIPreC can be used as a preventive measure for post-operative ischemic complications in known hospital settings. For example, prior administration of RIPreC to patients undergoing endovascular procedure can potentially reduce the high risk of ischemic or haemorrhagic stroke insult after surgical treatments for several clinical settings including intracranial aneurysms and carotid endarterectomy (26–29); (2) remote ischemic per-conditioning (RIPerC), which is applied during the ischemic event (prior to any recanalization); and (3) remote ischemic post-conditioning (RIPostC), which is applied after the ischemic event (i.e., following recanalization) or during reperfusion. The latter two conditioning paradigms have promise for translation, as they are non-invasive and can be administered pre-hospital (i.e., in an ambulance while transferring stroke victims to the emergency center) or following recanalization therapy (14).

## RIC Efficacy

Several parameters might affect the overall efficacy of RIC in reducing the infarct size following the focal ischemic stroke, including sex, age, animal species and different models of focal ischemia (30). A recent meta-analysis and systematic review has shown that there is no significant difference in RIC beneficial effects between reperfusion (e.g., intraluminal and embolism models) and permanent (e.g., cauterization, use of a permanent clip, permanent distal MCA ligation, permanent intraluminal suture) models of focal brain ischemia (30). However, RIC was shown to be more efficacious in male rodents relative to their female counterparts (30). As expected, older animals show significantly larger ischemic damage when compared with younger adult group due to several factors, namely, rarefaction of cerebral collaterals, decreased arteriole dimeter, higher tortuosity in cerebral vessel (31–33). All these factors affect the aged animals' ability to compensate for the poor blood flow circulation. On the other hand, the cellular and biochemical alterations associated with aging process, such as higher expression levels of pro-inflammatory cytokines and exacerbated oxidative stress, will reduce the cell survival rate in aged population and increases the neuronal cell injury and death (34, 35). Consequently, aged stroke groups may benefit less from the neuroprotective effects of RIC and may show more limited functional recovery in both pre-clinical and clinical settings (36).

## RIC: Underlying Mechanisms of Action

**Figure 1:**
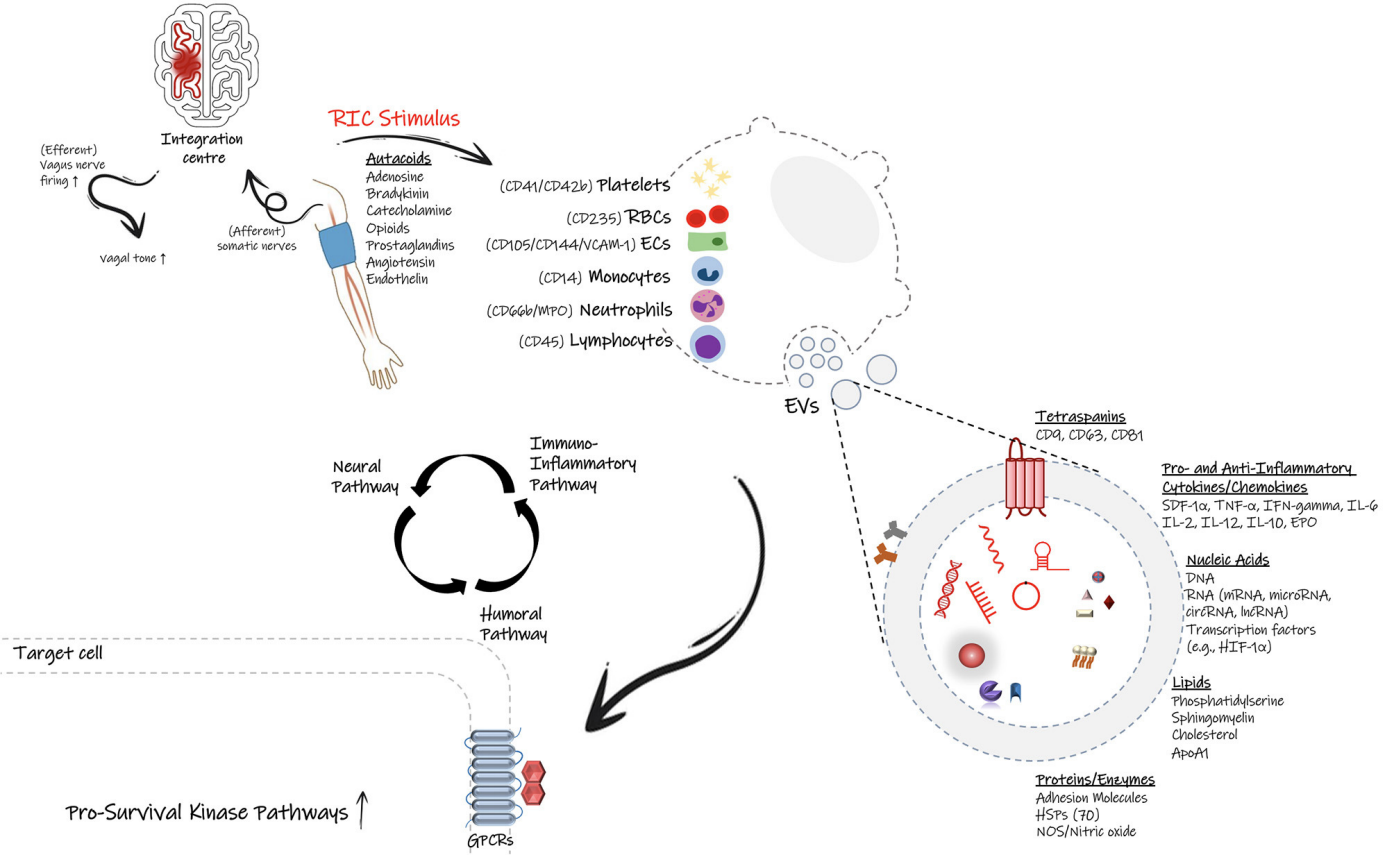
Proposed signal transmission pathways during remote ischemic conditioning (RIC). Following application of RIC protocol on the upper arm, the initiated protective signal appears to involve a complex and overlapping activation of neural pathways (autonomic nervous system), humoral mediators, and the peripheral immune system. It is proposed that small endogenous particles called extracellular vesicles (EVs) can facilitate the transfer of protective effects of RIC through the flow stream. Many cell types including endothelial cell, immune cells as well as platelets can generate EVs with signature surface markers and cargo defined based on the parent cell and the physio/pathophysiological conditions. EVs can carry cytokines, chemokines, genetic material and many more biological substrates, which allow them to inter-connect distant cells, tissues or organs and affect the target cells' transcriptional profiles and likely their function and phenotype. Based on the stimulus, they can deliver either proinflammatory or anti-inflammatory factors, therefore modulate the immune response and the fate of recipient cells.

Extensive research has been conducted in the preclinical and clinical settings to investigate the underlying mechanisms of RIC. Still, the primary molecular pathways are somewhat equivocal, possibly due to the contribution of several complex and overlapping signaling pathways. Although much of the research to date focuses on the protective role of RIC on cardiomyocytes in the heart, there is a growing focus on brain ischemia, and the underlying mechanisms of cardio- and neuro protection likely overlap. Multiple hypotheses have been proposed on how the protective signal is transferred from the periphery to the target organ. Generally, three pathways are thought to play a role in RIC protection (Figure 1).

### Neurogenic Pathway

Modulation of autonomic nervous system has been shown to play a key role in RIC-induced distant organ protection in both experimental and clinical studies (37–39). In a rat model of cerebral ischemia investigating the neurogenic mechanism led to the neuroprotective effects of RIPreC, pharmacological inhibition of autonomic ganglia with hexamethonium (a ganglionic blocker) reversed the reduction in cerebral infarct size in animals undergoing RIPreC, thereby indicating the potential role of neural pathways in relaying the protective signals generated by conditioning stimulus (37).

### Humoral Pathway

Following the conditioning stimulus, blood-borne molecules are released into the circulation and then travel from the remote site toward the target organ to exert their protective functions. The humoral nature of the conditioning signal is supported by several lines of evidence: First, every brief ischemic cycle is followed by a brief reperfusion cycle. This allows the factors secreted during the ischemic conditioning to flow in the bloodstream toward the target organ; Second, cross-individual blood transfer from a conditioned subject to an unconditioned control can confer the protection against injury in preclinical models (40). Recently, Pickard et al. reported that there is an interdependence between the neural and humoral pathways in mediation of cardioprotection following RIC (41). In other words, the secretion of circulatory factors may rely on the prior firing of the vagal nerves and stimulation of autonomous nervous system (41), and the humoral release of some factors may lead to the activation of sensory afferent nerves. For example, the release of autacoids at the site of remote ischemia may initiate neuronal and humoral signal transduction, and contribute to the protective effects of RIC (41). Prominent autacoids such as adenosine, bradykinin, catecholamines, opioids, and prostaglandins are secreted locally in the conditioned limb. Some autacoids can stimulate the afferent neural pathways, while others, such as nitric oxide and endothelin (ET), are mainly characterized by vasoactive effects on the blood vessels (14).

### Immune-Mediated Pathway

Neuroinflammation involves the activation and release of proinflammatory mediators from the brain resident immune cells (microglia and astrocytes) as well as the peripherally derived immune cells, such as neutrophils, monocytes, and T cells (42–44). Evidence suggests that RIC can inhibit not only the activation of microglia and astrocytes following an acute ischemic stroke but also the recruitment of circulating peripheral immune cells into the ischemic brain (45). Several studies have shown that RIC can reduce the infiltration of leukocytes in the brain, and therefore alleviate the inflammatory status in the brain. Considering the integral presence of leukocytes during cerebral ischemia, modulation of leukocyte gene expression by RIC is probably inevitable (46); however, limited studies have focused on the regulatory effects of RIC on leukocyte gene transcription (46). In the setting of cerebral ischemia, emerging evidence suggests systemic immune cell responses change during RIC-mediated neuroprotection, which will be further discussed later in this review.

## RIC: Collateral Blood Flow Enhancement

In addition to direct effects on target organs, RIC has direct effects on improving blood flow in vulnerable tissue. Preclinical stroke studies in mice suggest that RIPerC is effective alone and in combination with i.v. r-tPA in enhancing penumbral flow in young male mice, ovariectomized female mice, and 12-month old male mice (14, 47–49). Remote ischemia has also been associated with increased cerebral blood flow in humans (50–54).

The collateral circulation is a key determinant of infarct progression in AIS. Good collateral flow is associated with reduced infarct expansion and better stroke outcome (55–64). However, a progressive constriction of collateral arterioles over time after ischemic onset may contribute to infarct growth (36, 65, 66). RIC may improve collateral flow by preventing narrowing of key collateral vessels, and is associated with improved collateral flow and reduced infarct in preclinical studies (4, 65, 67–69). Thus, preventing collateral failure is thus critical to improve outcome in stroke patients, and RIC may improve collateral flow. However, the exact mechanisms of enhanced collateral flow due to RIC are not defined.

RIC may increase cerebral blood flow (CBF) either through formation of new vascular branches (angiogenesis and/or arteriogenesis) or strengthening of the existing vasculature. Some of the major signaling mediators of CBF enhancement are discussed below.

### The eNOS/NO/Nitrite System

Nitric oxide (NO) is a key regulator of vascular tone and blood flow in the brain (70). NO is primarily generated *via* enzymatic function of three types of nitric oxide synthase (NOS), namely endothelial NOS (eNOS), neuronal NOS (nNOS), and inducible NOS (iNOS) (70). Following an ischemic insult, nNOS is activated soon after elevation of intracellular Ca2+ levels and produces NO to regulate cerebral vascular tone and blood flow (71). Afterwards, NO derived from eNOS in vascular endothelium contributes to flow-mediated vasodilation (71). Available evidence suggests that NO released during a brief period of ischemia (produced by nNOS and eNOS) may play a neuroprotective role against prolonged focal ischemia, mainly through formation or strengthening of collateral vessels in order to maintain the cerebral microcirculation, as well as preventing platelet aggregation. In addition, NO is the main driver of blood flow through the collateral circulation toward the site of injury (71). By contrast, large amount of NO produced by iNOS is associated with neurotoxic effects, such as lipid peroxidation, reaction with superoxide (O2•-) to form peroxynitrite (OONO–), and protein nitrosylation. Nevertheless, iNOS is only expressed when it is induced by proinflammatory factors (70).

In the field of stroke research, several studies have demonstrated RIC neuroprotection has been associated with collateral flow enhancement. For instance, in a rat model of chronic cerebral hypoperfusion (CCH, bilateral common carotid arteries were ligated), RIC treatment (3 cycles of 8-min occlusion/release of bilateral hindlimbs, for 28 days) significantly augmented cerebral perfusion measured by laser speckle contrast imaging (LSCI) at day 14 after CCH onset, compared to non-treated group (72). The results were associated with an increased number of vessels in hippocampus, and accordingly a better learning capacity and spatial memory ability in RIC-treated rats. Mechanistically, based on western blot data RIC caused neuroprotection through preservation of eNOS activity (i.e., by promoting neovascularization in hippocampus); however, a NOS inhibitor (L-NAME) abolished all the RIC protective effects (72). As mentioned earlier, NO is primarily derived from eNOS; moreover, Nitrite can also be a prominent source of NO, which circulates in the bloodstream with RBC/hemoglobin and it is reduced to NO, especially in response to ischemic insult to mediate vasodilation (73). In a mouse bilateral CCAO model, Hess and coworkers noticed a dramatic increase in plasma nitrite after 2 weeks of daily RIPostC treatment, 4 cycles of 5 min occlusion/reopening of both hindlimbs, started 7 days after bilateral CCAO, and rise in nitrite level was correlated with augmented CBF (47). Altogether, literature suggests that NO and nitrite are two signaling molecules that play a key role in RIC mechanism of neuroprotection, in particular *via* strengthening the collateral vessels and maintenance of cerebral microcirculation (47, 72).

### Notch Signaling Pathway

There is also evidence that stimulation of Notch1 signaling pathway by RIC can mediate neuroprotection. Preclinical experiments in rats with focal cerebral ischemia demonstrated a higher rate of arteriogenesis in brain sections from RIC (RIPerC + RIPostC)-treated rats, as indicated by increased arterial diameter and more proliferative (BrdU+) smooth muscle cells in peri-ischemic core when compared to non-treated MCAO rats (74). Additionally, RIC improved local CBF on the cortical surface supplied *via* leptomeningeal collateral anastomoses. Increased arteriogenesis induced by RIC was correlated with activation of Notch 1 signaling, as the expression of Notch receptor and its intracellular domain (NICD) was significantly elevated in ischemic arteries by RIC (74). Therefore, RIC-induced arteriogenesis and increased cerebral perfusion through enhanced collateral branches can be somewhat attributed to the activation of Notch signaling pathway (74).

### VEGF/VEGF Receptor Signaling Pathway

Vascular endothelial growth factor (VEGF) signaling is another possible mechanism underlying the effect of RIC on cerebral blood flow. VEGF is known to modulate vascular tone after binding to its corresponding tyrosine kinase receptor on the vascular endothelium thereby promoting the release of vasodilatory compounds such as prostacyclin and NO (75, 76). Elevated levels of NO in response to VEGF binding to VEGF receptor type 2 (VEGFR2), can induce angiogenesis and regulate the endothelial function and migration (76). Some studies have identified that VEGF mRNA and protein expression level is up-regulated following RIC treatment (77, 78). In a mouse model of spinal cord ischemia, ischemic preconditioning applied to the abdominal aorta (3 × 5 min of alternative clamping and reperfusion) resulted in high VEGF protein levels in plasma, with a resultant neuroprotective effect (78). Although early increase in VEGF/VEGFR expression can cause BBB permeability and exacerbate the ischemic injury, later up-regulation at the border of ischemic core can increase the number of capillaries (neovascularization) and restore the cerebral microvascular circulation after stroke (79).

## RIC: Regulation of Cell Survival and Apoptosis Signaling

**Figure 2:**
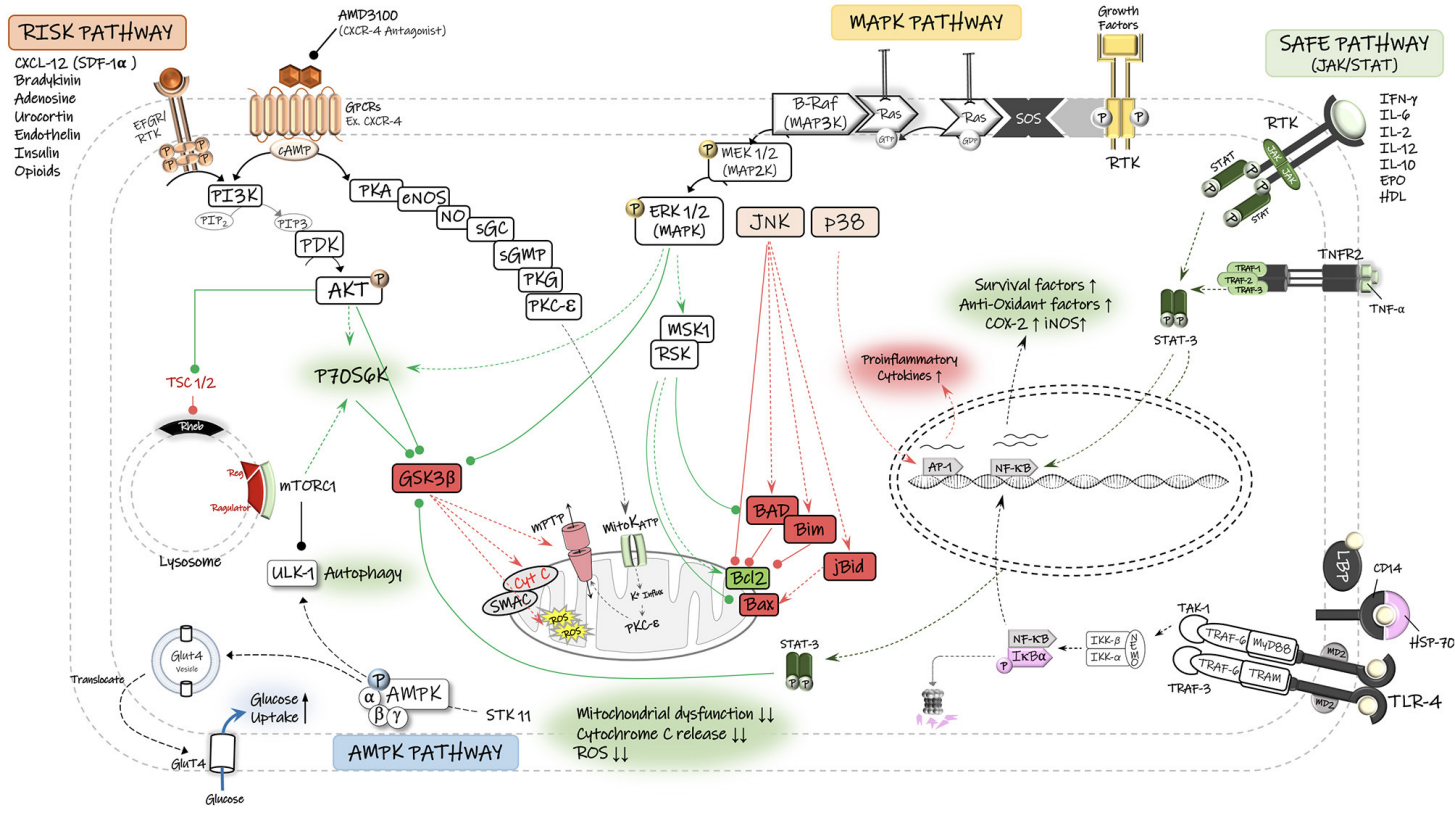
Schematic diagram of the potential signaling pathways involved in neuroprotective effects of remote ischemic conditioning (RIC). The reperfusion injury salvage kinase (RISK) pathway and its major pro-survival kinases, including PI3-K, phosphatidylinositol 3-kinase; PDK, phosphoinositide-dependent protein kinase; AKT/PKB, protein kinase B. The survivor activating factor enhancement (SAFE) pathway and its major signaling components, including STAT, signal transducer and activator of transcription, JAK, Janus kinase. The Ras/Raf/Mitogen-activated protein kinase/ERK kinase (MEK)/extracellular-signal-regulated kinase (ERK) cascade is activated through ligand binding to surface receptor. MAPK signal transduction results in activation of mitogen and stress activated kinase 1 (MSK1) and pp90 ribosomal S6 kinase (RSK), thereby leading to inactivation of the pro-apoptotic protein Bad and up-regulation of anti-apoptotic proteins of the Bcl-2 family. There is also an interplay between MAPK and RISK pathways that together with SAFE pathway converge on the mitochondrial permeability transition pore (mPTP) and prevent its opening and the consequent release of cytochrome C (Cyto C) and reactive oxygen species (ROS) into the cytosol. Under stress (Ischemia/hypoxia) conditions and low energy levels (↑AMP:ATP), the cell energy sensor adenosine monophosphate-activated protein kinase (AMPK) gets phosphorylated and activated by its upstream serine/threonine kinase 11 (STK11) and enhances the translocation of glucose transporter GLUT4 to cell membrane. AMPK-dependent autophagy contributes to functional recovery and neuroprotection. Autophagy is also regulated by the mammalian target of rapamycin (mTOR) complex-1. TNF-α, tumor necrosis factors-alpha; TNFR2, tumor necrosis factors receptor type-2; RTK, receptor tyrosine kinase; mitoKATP, mitochondrial ATP-sensitive potassium channel; GSK3β, glycogen synthase kinase 3 β; GPCR, G protein-coupled receptor; P70S6K, p70 ribosomal S6 protein kinase; PKA, protein kinase A; PKC, protein kinase C; PKG, protein kinase G; cGMP, cyclic guanosine monophosphate; NO, nitric oxide; Ulk, autophagy-initiating kinase; TSC, tuberous sclerosis complex; Rheb GTPase, direct mTORC1 activator; AP-1, activator protein-1; JNK, c-Jun N-terminal kinase; NF-κB, nuclear factor kappa B; TLR-4, toll-like receptor-4.

RIC increases tolerance and viability of brain tissue during cerebral ischemia by activating signaling that supports survival and inhibits apoptosis, and by reducing inflammation. Several protective signaling pathways and pro-survival kinases and mediators have been shown to be involved in RIC-induced protection (Figure 2). The two most widely studied pathways are (1) the reperfusion injury salvage kinase (RISK) pathway, with its major signaling *via* Akt and Erk1/2, and (2) the survivor activating factor enhancement (SAFE) pathway, with its major signaling *via* Janus Kinase (JAK) and signal transducer and activator of transcription 3 (STAT-3) (15, 80–83). These signaling pathways can be triggered by a variety of factors, including SDF-1α, MIF, HIF-1α, heat shock proteins (HSPs), nitric oxide (NO), mammalian target of rapamycin, MMPs, adenosine, bradykinin, erythropoietin (EPO), endocannabinoids, and tumor necrosis factor-α (TNF-α) (15, 80, 84), and are discussed in greater detail below. Furthermore, AMPK signaling pathway is increasingly recognized as a potential mediator of cell survival following RIC, and is discussed below (85–88).

### Risk Pathway

The RISK pathway is a possible protective signaling cascade through which RIC may exert its protective effects against reperfusion injury, *via* the activation of pro-survival kinases in two parallel signaling cascades—phosphoinositide-3 kinase (PI3K)/Akt and MEK1/2-ERK1/2 (80, 81, 83, 89). The RISK pathway was described by Schulman et al. when examining the cardioprotective potential for the growth factor urocortin on both isolated and *in vivo* models of acute myocardial infarction in adult rats (89). Urocortin is a peptide which belongs to the corticotropin-releasing factor (CRF) family and can regulate the mitogen activated protein kinases (MAPK)/extracellular signal-regulated kinases (ERK) pathway (89, 90). A significant reduction in the myocardial infarct size following the urocortin administration at reperfusion has been reported, which was associated with a notably higher levels of phosphorylated (ERK1/2) MAP kinase. This protection was not altered by the inhibition of other subfamilies of MAP kinases, p38 MAPK and Jun N-terminal kinase (JNK), suggesting that ERK1/2 (MAPK1) is responsible for the cell survival, while p38 and JNK are part of the death pathway in the ischemic setting (89). In a rat model of neonatal hypoxia-ischemia (HI), RIPostC reduced cerebral infarct size in an opioid-mediated activation of PI3K/Akt/Bax signaling pathway, as pharmacological inhibition of PI3K (*via* wortmannin) or opioid receptor (*via* naloxone) decreased the phospho-Akt expression levels and abrogated the infarction reduction and improved neurological outcomes achieved by RIPostC treatment. The serine/threonine kinase Akt (also known as protein kinase B) acts as an effector protein of PI3K pathway and it phosphorylates several downstream targets, including GSK-3β, Bad, and Bax (91). Akt-induced phosphorylation of Bax at Ser-184 reduces its half-life and inactivates its insertion into mitochondrial membranes, therefore blocking Bax-mediated proapoptotic pathways (91).

Activation of the RISK pathway following the ischemic conditioning, and phosphorylation of downstream effectors is believed to cause tissue protection *via* preventing mitochondrial permeability transition pore (mPTP) and inhibiting the release of cytochrome C into the cytosol and thereby activation of caspases and apoptosis will not be initiated (80).

### Safe Pathway

Another signaling pathway involved in RIC-induced protection is the survivor activating factor enhancement (SAFE) pathway, with the participation of key proteins Janus Kinase 2 (JAK2) and the signal transducer and activator of transcription (STAT-3 and−5) (81–83). As demonstrated by Heusch and colleagues, RIPreC application in patients undergoing coronary artery bypass surgery increased the phosphorylated form of STAT-5 (pSTAT-5) in myocardial biopsies (92). In agreement, treatment of isolated rat and mice hearts (93) with either Tryphostin AG490 (JAK Inhibitor) or PPI (Src kinase blocker), which both inhibit the phosphorylation of STAT5a *via* the upstream kinases (JAK and Src kinase), abolished preconditioning-mediated cardioprotection. Moreover, preconditioning protection cannot be achieved in STAT5a-deficient (knock-out) mice (93) and genetic depletion of functional STAT-3 in mice cardiomyocytes prevented preconditioning-induced protection against the ischemic injury (94). In a pig myocardial IR injury model, Heusch et al. demonstrated that RIPostC activates the mitochondrial STAT-3 in the heart that preserves the function of mitochondria in cardiomyocytes and confers cardioprotection against IR injury, and pharmacological inhibition of STAT-3 abolished the effects (95).

There is evidence that SAFE and RISK pathways can confer tissue protection independently from each other. Lecour et al. reported that “pharmacologic” preconditioning of rat hearts (subjected to 30-min regional IR injury) with a low dose of TNF-α injection confers the same cardioprotective properties as “ischemic” preconditioning (96). The activation of TNF-α receptors triggers phosphorylation of STAT-3 by either JAKs or MAPKs and initiation of cell survival pathways (96). In this study, despite blocking different components of RISK pathway, including PI3K (*via* wortmannin), MAPK-Erk1/2 (*via* PD-98059), and mTOR (*via* rapamycin), STAT-3 expression did not change and cardioprotection achieved by TNF-α preconditioning was not abrogated, indicating the independent function of SAFE pathway (96).

Conversely, Tamareille et al. indicated that there is a crosstalk between the RISK and SAFE pathways in RIPerC alone, local IPostC (with conditioning and ischemia in the same target organ) alone, and combined RIPerC + IPostC in rat myocardial IR injury model (83). They confirmed this interaction since cardioprotective effects against reperfusion injury were fully abrogated *via* the pharmacological inhibition of either RISK (with wortmannin, an inhibitor of PI3 K/Akt signaling pathway, and with U0126, an inhibitor of MEK1/2) or SAFE (with AG490, an inhibitor of JAK/STAT pathway) (83). In other words, inhibitors of RISK abrogated the phosphorylation of STAT-3, and inhibitor of SAFE (AG490) blocked the phosphorylation of survival kinases from RISK (Akt, ERK1/2, and GSK-3β) pathway (83).

Emerging evidence indicates that these pro-survival signaling pathways (SAFE and RISK) potentially converge on the mitochondrial permeability transition pore (mPTP), high-conductance channel proteins located in the mitochondrial inner membrane important in cell death signaling (97, 98). In case of excessive calcium entry or high ROS exposure under ischemia/hypoxia conditions, the opening of mPTP allows the release cytochrome C into the cytosol and that can lead to the cell death (99). Therefore, inhibition of mPTP opening supports cell survival under pathologic conditions (99). The cytoprotection received through the phosphorylation of key prosurvival kinases of RISK (Akt, Erk1/2, GSK-3β) and SAFE (STAT3) pathways is dependent on the inhibition of mPTP, suggesting a key protective role for this pathway in molecular signaling induced by RIC (97).

### AMPK Pathway

Compelling evidence has suggested that AMPK signaling can contribute to RIC-mediated neuroprotection against the cerebral IR injury (88). AMPK (5'-AMP-activated protein kinase) is a member of the serine/threonine (Ser/Thr) kinases and an early energy sensor that responds to stressful stimuli such as ischemia/hypoxia and energy deprivation (100). Under low energy conditions, higher activation of AMPK signaling pathway contributes to elevated glucose uptake and utilization in neurons. We have recently shown that RIPerC-mediated neuroprotection and collateral flow enhancement in a rat model of focal ischemia is associated with an increase in pAMPK/eNOS activity (86). AMPK is considered to be a direct activator of eNOS/NO system. Hence, the improved cerebral blood flow in RIPerC-treated rats can be attributed to AMPK-mediated eNOS activation and NO production, resulting in vascular relaxation and flow increase (86, 101).

There is also evidence that AMPK reduces the ischemic injury by triggering autophagy (catabolic) pathways in several organs, including heart (102–105) and kidney (106). However, the extent to which AMPK-induced autophagy plays a protective or destructive role in conditions of cerebral ischemia is unclear (107, 108). In general, autophagy serves as a prosurvival/cytoprotective mechanism during metabolic stresses and protects the cell through degradation of damaged organelles and aggregated proteins into basic biomolecules, which are then recycled for energy regeneration (107). Up-regulated autophagy processes through AMPK-related signaling have been associated with suppressed neuronal apoptosis and alleviated cerebral ischemic damage (88). In a mouse model of cerebral ischemia, RIPostC, applied *via* 3 cycles of 10-min occlusion/reopening of bilateral femoral arteries at the time of reperfusion following 2 h MCAO, was associated with improved neurological outcome as well as a smaller infarct size (88). However, neuroprotective effects of RIPostC were abolished when mice were given the autophagy inhibitor 3-methyladenine (3-MA) prior to RIPostC treatment and partially abolished when mice received compound C, an AMPK inhibitor, indicating RIPostC mediated neuroprotection *via* activation of AMPK-dependent autophagy (88). In addition, anti-apoptotic properties of RIPostC were abrogated by 3-MA treatment, as indicated by up-regulation of apoptotic agents like Bax and caspase-3, and downregulated anti-apoptotic Bcl2 (88). Liu et al. demonstrated that metformin-treated mice had reduced brain injury after 90-min MCAO (109). Metformin is a glucose-lowering medication for type 2 diabetes (110) that can protect against the inflammation and endothelial dysfunction associated with the cerebral ischemia reperfusion injury through the activation of AMPK signaling pathway (109). Metformin alleviates cerebral I/R injury through activation of AMPK-dependent anti-inflammatory mechanisms including AMPK-induced suppression of NF-κB pathway, reduced expression of proinflammatory cytokines (IL-1β, IL-6, TNF-α) and adhesion molecules (ICAM-1), reduced neutrophil infiltration, and reduced endothelial injury and BBB permeability (109).

## RIC: Humoral Mediators

The alternative hypothesis to the “signal transfer through the activation of neural pathway” is that the protective signal generated locally in the remote site (like a limb) may have humoral (blood-borne) nature. Several blood-borne mediators have been identified as important for protection *via* RIC. These factors can travel *via* the circulation toward target tissue, wherein they can modulate inflammation and cell death.

### SDF-1α

The chemokine stromal-derived factor-1 alpha (SDF-1α, also termed as CXCL12) is one of the most studied humoral factors involved in cerebral and cardiac ischemic conditioning (84, 111–113). It is classified as an atypical cytokine that binds to the G-protein-coupled CXCR4 receptor, which has an abundant expression on the endothelial cells (114). SDF-1α binding to CXCR4 activates several down-stream signaling pathways (115) including the Gi-protein/Src/PI3K-Akt-NF-κB and the PKC pathways that activate the Ras/Raf/MAPK axis (115). Activated MAPK p42/44 (Erk1/2) will in turn translocate into the nucleus and mediates transcriptional activation (115) (Figure 2). Activation of JAK proteins and recruitment of STAT transcriptional factors may also be associated with the activation of SDF-1α-CXCR4 axis in some cell types (116). SDF-1α stimulation can lead to the activation of nuclear factor (NF)-κB, well-known for its role in inflammation (115). Following translocation into the nucleus, NF-κB binds to specific DNA sequences in the promoter region of critical mediators of immune and inflammatory responses and regulates their gene expression levels (117). Activated NF-κB also enhances the expression of anti-apoptotic target genes (118, 119) and the expression of target genes that encode for antioxidant proteins, reducing necrotic cell death (119).

Of particular relevance to the ischemic conditioning-induced tolerance is the finding that preconditioning treatment with SDF-1α could induce the activation of antiapoptotic pathways and protect cardiac myocytes from hypoxia/reoxygenation damage (112). Hu et al. reported that infusion of SDF-1α into the left ventricular cavity of mice prior to 30 min of LAD coronary artery occlusion enhanced cell survival equivalent to ischemic preconditioning (112). Ischemic PC, which was applied *via* 6 cycles of 4-min occlusion/reopening of coronary artery, was associated with a 4.5-fold increase in SDF-1α mRNA transcript level and a significant reduction in the myocardial infarct size. Incubation of isolated mice cardiomyocytes with SDF-1α significantly increased the phosphorylation of Erk and Akt within 5 min; conversely, JNK and p38 phosphorylation sharply declined (112). Notably, pretreatment of cultured myocytes with specific antagonist of CXCR4 (AMD3100) prior to SDF-1α exposure prevented the protective effects of SDF-1α on myocyte survival, suggesting SDF-1α-CXCR4 binding mediates anti-apoptotic mechanisms of preconditioning (112).

### MIF

Macrophage migration inhibitory factor (MIF) is another putative candidate for the “remote signal” in the conditioning paradigm (120–122). MIF is a pleiotropic chemokine-like inflammatory cytokine that acts as a key regulator of innate and adaptive immunity states such as neuroinflammation and the stress response (123). In response to proinflammatory stimuli, MIF is released from cytosolic pools of almost all types of immune cells into the circulation rather than being rapidly upregulated at the transcriptional levels (123). MIF can engage several different receptors as well as intracellular binding partners, and thereby exert varied biological functions (123). MIF can not only bind to its cognate cytokine receptor (CD74), but also it can be a non-cognate ligand for the CXCR2 and CXCR4. CXCR2 is dominantly found on the surface of neutrophils and monocytes/macrophages, while CXCR4 is ubiquitously distributed on many cell types (123, 124). There is evidence that MIF binds to CXCR2 and 4 with high affinity and provokes the recruitment of leukocytes to the site of inflammation (125, 126). Of note, MIF binding to CD74 can initiate the formation of a functional complex comprising CD74, proteoglycan CD44 and Src kinases, which then leads to the sustained activation of the Erk1/2 MAPK pathway (127), giving rise to different regulatory effects on expression of downstream transcription factors including Elk-1, AP-1, and cMyc (127). MIF can also interact with intracellular binding proteins, such as JUN-activation domain-binding protein 1 (JAB1), through which it can block the activity of JNK pathway and its target transcription factor AP-1 (128). Recent studies implicate that MIF may play a mediatory role in conditioning-induced protection (120, 121). In an animal study of ischemic heart disease, ischemic PC (3 cycles of 5-min myocardial ischemia/reperfusion) mediated cardioprotection against the prolonged 60 min ischemia/3 h reperfusion injury in wild-type (WT) mice (120). However, ischemic PC had no protective effects in mice with MIF-knock out (MIFKO), suggesting that MIF released from the preconditioned myocardium mediates the PC protection. Notably, PC lowered the density of infiltrated inflammatory cells [by 35% in CD45+ cells (leukocytes) and 63% in CD68+ cells (macrophages)] in the WT, but not in MIFKO hearts (120). Western blotting data revealed a noticeable increase in phosphorylation and activation of RISK and AMPK signaling components in PC-treated WT hearts. PC significantly increased p-Erk1/2, p-Akt, p-p70S6K, and p-GSK3β (proteins involved in RISK pathway), AMPK phosphorylation, cell-surface GLUT-4 translocation and glucose uptake. MIF deficiency abrogated all these effects of PC, indicating that MIF is exerting its role through activation of RISK and AMPK pathways (120).

### ApoA1

Apolipoprotein (ApoA1) is the main structural constituent of high-density lipoproteins (HDL). The plasma levels of HDL cholesterol is inversely correlated with the risk of cardiovascular diseases (CVDs) (129), meaning that patients with low HDL levels are more prone to CVD incidence, particularly to the formation of atherosclerotic plaques. Clinical strategies to increase HDL levels can lower the risk of CVDs (129, 130). In addition to anti-atherogenic properties of HDL and ApoA1, both have proven to be protective by modulating anti-inflammatory, anti-oxidative, and antiapoptotic pathways. Moreover, compelling evidence suggests that ApoA1 may act as a humoral mediator of RIC through induction of pro-survival signaling pathways. The plasma levels of ApoA1 have been shown to be upregulated in response to RIC in animals and humans (131, 132). Kalakech et al. suggested that ApoA1 may be involved in the protection conferred by RIC, based on data showing i.v. administration of ApoA1 prior to prolonged myocardial ischemia (MI, 40-min occlusion of coronary artery in rats) could mimic cardioprotection achieved by RIPreC (81). ApoA1-treated rats *in vivo* showed a significant reduction in myocardial infarct size along with an increase in phosphorylation and activation of RISK and SAFE signaling, including Erk1/2, Akt, and GSK3β. However, pretreatment with either Wortmannin (a PI3K/Akt pathway inhibitor) or U0126 (MEK1/2-ERK1/2 pathway inhibitor) prior to ApoA1 administration markedly abolished the cardioprotective effects of ApoA1 (81) (Figure 2). Furthermore, acute injection of ApoA1 exhibited anti-inflammatory properties, including lower infiltration of leukocytes to the infarcted area, downregulated adhesion molecules (e.g., ICAM-1) and hence lower leukocyte-EC interaction and adhesion, lower expression of pro-inflammatory cytokines (e.g., TNF-α and IL-6). Taken together, experimental and clinical data suggests that ApoA1 may contribute to the RIC protection during MI through activation of anti-apoptotic proteins and modulation of inflammatory response.

### TNF-α

Tumor necrosis factor-alpha (TNF-α) has been identified as an essential contributor to the induction of ischemic tolerance. TNF-α is small (17 kDa) inflammatory cytokine produced by macrophages, monocytes, neutrophils, mast cells, T and B lymphocytes upon stimulation (e.g., ischemic injury) during acute phase of inflammation (133).

Intriguingly, the function of TNF-α in the cerebral ischemia and ischemic conditioning is controversial. Pathophysiological levels can not only compromise the integrity of BBB and exacerbate the inflamed brain injury, but also can activate the pro-apoptotic factors and caspases and cause cell death. On the other hand, genetic deletion of TNF receptors in mice prior to focal stroke has shown to increase neuronal cell death because of higher oxidative stress and suppressed microglial reactivity, implicating TNF-α as a neuroprotectant in ischemic brain (134).

TNF-α can exert pleiotropic effects by signaling through two types of TNF receptors, either TNFR1 or TNFR2. TNF-α binding elicits complex signaling cascades that varies according to receptor subtype. Given the neurotoxic and neuroprotective signaling elicited by TNFR1 and TNFR2, respectively, the ratio of TNFR1:TNFR2 may be a key determinant in TNF-α overall effects (135). There are two bioactive forms of TNF: transmembrane TNF (tmTNF) and soluble TNF (solTNF) (133, 136). While tmTNF can activate both TNFR1 and TNFR2, solTNF can only signal through TNFR1 that is widely expressed on almost all cells (133, 136). TNFR1 activation triggers the recruitment of TNFR1-associated death domain protein (TRADD), which in turn can initiate two different signaling cascades regulating both cell survival and apoptosis (133, 136, 137). TNF-α can confer resistance to cell death through formation of protein complex I, where TRADD recruits TNFR-associated factor 2 (TRAF2) and leads to the stimulation and activation of NF-κB transcription factor. Translocation of NF-κB into the nucleus can promote the transcription of protective genes, including antioxidant enzyme Mn-superoxide dismutase (Mn-SOD) and calcium chelator calbindin (135). Therefore, TNF-α can modulate reduction of reperfusion injury by binding to TNF receptors and triggering the upregulation of antioxidant activity through NF-κB-dependent dismutase Mn-SOD synthesis. Alternatively, TRADD can induce programmed cell death *via* formation of complex II through interaction of Death Domain (DD) sequence of TNFR1 with Fas-associated death domain protein (FADD) and caspase 8 (138). Unlike TNFR1, TNFR2 does not contain DD and its expression is confined to regulatory T cells (Tregs), endothelial cells and some subset of cells in CNS. TNFR2 directly engages TRAF2 and activates pro-survival (PKB/Akt) and NF-κB pathways (139).

Several preclinical and clinical studies have provided evidence supporting the neuroprotective role of TNF-α and its upregulation following the conditioning stimulus (140–142). Therefore, mild elevation of TNF-α during ischemic conditioning can induce protective properties by neutralizing the oxidative insult and enhancing cellular defense mechanisms against severe ischemic attack (137, 143).

## RIC: Immune-Mediated Neuroprotection

**Figure 3:**
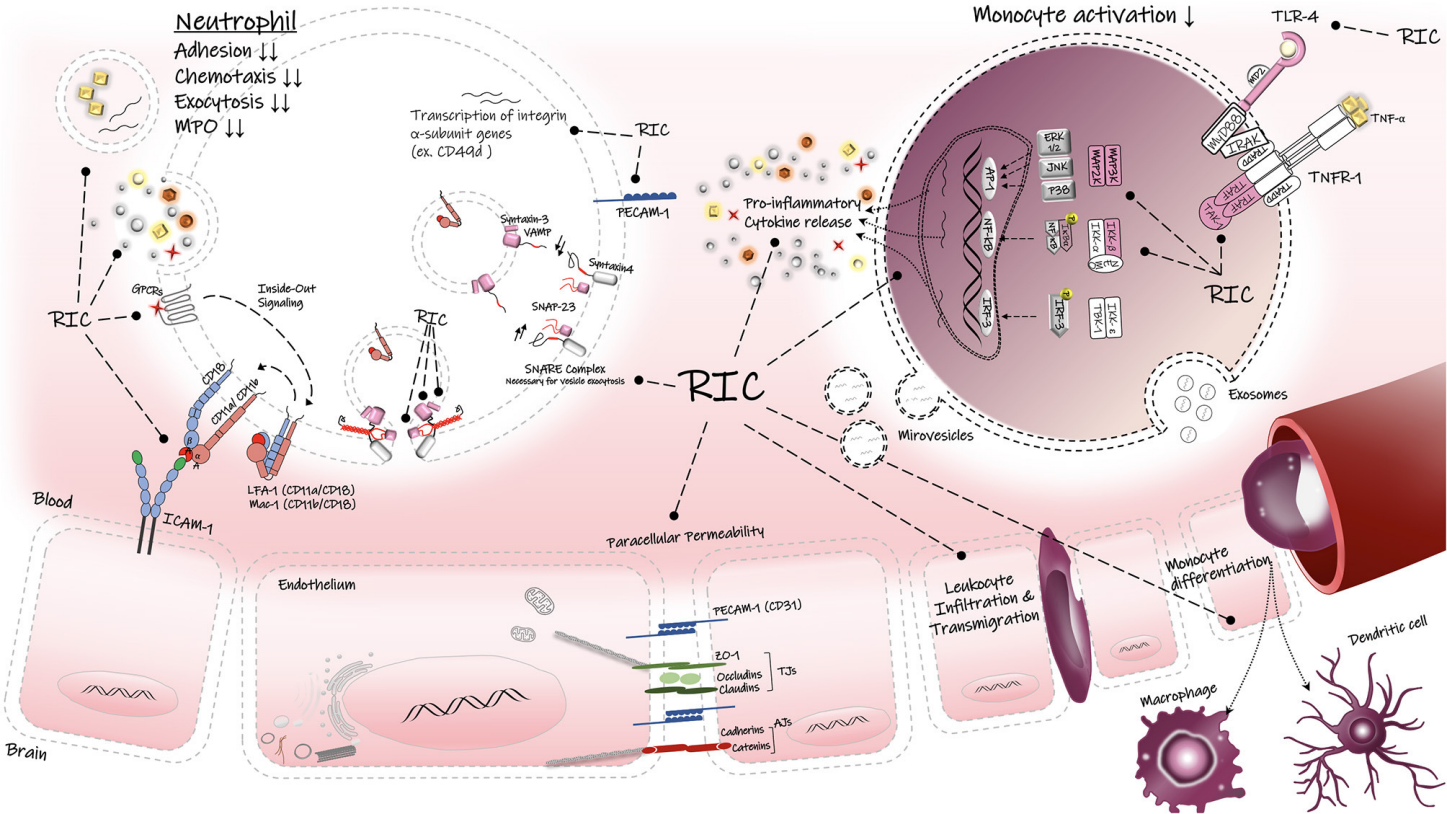
Regulatory effects of RIC on inflammatory responses. RIC can decrease the peripheral immune activation, infiltration and transmigration into the brain parenchyma. RIC can suppress the TLR-4/NF-κB associated proinflammatory cytokine release in the immune cells. LFA-1, lymphocyte function-associated antigen-1; Mac-1, macrophage-1 antigen; ICAM-1, intercellular adhesion molecule-1; PECAM, platelet endothelial cell adhesion molecule-1; Z0-1, zonula occludens-1; AJs, adherens junctions; TJs, tight junctions, TNF-α, tumor necrosis factors-alpha; TNFR1, tumor necrosis factors receptor type-1; TLR-4, toll-like receptor-4; SNAP-23, synaptosomal-associatedprotein-23; VAMP, vesicle-associated membrane protein; GPCRs, G protein-coupled receptor; TAK1, transforming factor-β-activated kinase 1; Iκβα inhibitor kappa beta kinase.

Preclinical and clinical evidence suggests that RIC confers neuroprotection in the setting of AIS (144). A growing number of studies are suggesting part of this effect might be due to differential responsiveness of peripheral circulating immune cells following the conditioning stimulus (140, 145). The immune response to ischemic conditioning is itself composed of molecular, cellular, and systemic mediators that may play a role in conditioning tolerance (145). The conditioning stimulus can prime the brain in advance by mobilizing both innate and adaptive immune responses so that by the time severe IR injury happens, the brain enters the “resolution of inflammation” or “recovery phase.” Thus, immunomodulation may contribute to conditioning-induced protection in brain and heart (140, 145–147) (Figure 3).

Several studies have demonstrated that molecular and cellular profile of inflammation change following the conditioning stimulus (140). In a rat model of focal ischemia, Liu et al. reported that RIPreC-mediated neuroprotection was associated with altered immune cell populations and cytokine profiles (140). In this study, a shift in the phenotype of splenic monocytes toward less or non-inflammatory (non-classical) monocytes (CD43^+^/CD172a^+^) was observed in the RIPreC-treated rats (140). Monocytes with inflammatory phenotype (classical monocytes) can infiltrate the brain and lead to highly inflammatory type of cell death *via* their potent ability to secrete inflammatory mediators and free radicals and to differentiate into macrophages and dendritic cells (148). Thus, RIPreC-mediated change in favor of non-inflammatory monocytes prior to focal stroke has been beneficial against ischemic attack (140). In addition, the expression levels of proinflammatory cytokines such as TNF-α and IL-6 were significantly elevated by RIPreC, suggesting that conditioning induced manipulation of the immune response may be a key mechanism of protection (140) (Figure 3).

Beside the importance of peripheral immune cells in conditioning effect, the brain's resident immune cells, microglia and astrocytes, are also considered to be cellular mediators of conditioning stimulus, as they contribute to resolution of neuroinflammation by promoting immunosuppression (145). These cells can release anti-inflammatory cytokines (e.g., TGFβ and IL-10) to inhibit the inflammatory response. In addition, astrocytes and neuronal cells arrange the “repair and regeneration” phase by producing growth factors such as insulin-like growth factor (IGF) and vascular endothelial growth factor (VEGF) that promote neuronal sprouting and angiogenesis (145).

Microglia exhibit a high level of TLR4 expression on their cell surface which helps them to initiate the innate immune response. While hyperactivation of these receptors are known to aggravate the inflammatory status, partial activation during a brief ischemic conditioning may confer neuroprotection by priming the brain against severe and prolonged ischemia (149). Pradillo et al. reported that prior exposure to ischemic preconditioning (IPC, 6-min occlusion of bilateral common carotid arteries) can induce immunological tolerance and therefore protect against the permanent MCAO in wildtype (WT) mice with normal TLR4 expression (149), as shown by a better neurologic outcome and reduced infarct size. However, genetic deletion of TLR4 receptors abolished the protective properties of IPC, indicating the importance of TLR4 for activation of innate immunity and induction of ischemic tolerance by IPC (149). They further observed IPC upregulated the protein levels of TNF-α, iNOS, and COX-2, p65 subunit of NF-κβ transcription factor and downregulated inhibitory kappa B alpha (IκBα). These molecular proteins have been suggested to mediate the ischemic tolerance by IPC (42, 149). Likewise, all the results were reversed in TLR4-deficient mice. Taken together, TLR4 signaling pathway mediates the IPC-induced neuroprotection *via* activation of transcription factor NF-κβ and therefore upregulation of TNF-α, iNOS, and COX-2 (149, 150).

Considering the fact that peripheral and resident immune cell activation can dramatically change the inflammatory status during and after the cerebral ischemia, RIC's ability to modify the immune response and thereby improve the stroke outcome poses this novel treatment as a potential therapeutic adjunct to already approved stroke therapies.

## RIC: Transcriptional Alterations In Circulating Leukocytes

Gene expression in circulating peripheral immune cells rapidly changes after AIS, and several studies have utilized gene expression profiling to investigate transcriptomic alterations in these cells (151–153). Genes differentially expressed after stroke in humans including neutrophils and monocytes (151, 152), suggesting neutrophils and monocytes play key roles in the genomic responses of circulating blood cells to AIS (151, 152, 154). Alterations in genomic patterns happens at an early stage (<3 h) after stroke onset, and these rapid changes can be used to make an early diagnosis of AIS in humans (151). Notably, neutrophils are the first immune cells to arrive at ischemic brain tissue and are key contributors to BBB permeability, cerebral edema and brain injury (154). Therefore, therapeutic approaches that target the deleterious aspects of neutrophil activation, including neutrophil-mediated BBB disruption, neutrophil transmigration and infiltration, and their interaction with the neurovascular unit (NVU), may be helpful to reduce brain edema and therefore improve the stroke outcome (155).

The significance of transcriptional gene screening of blood cells lies in its potential to identify and validate specific genes as molecular biomarkers in ischemic stroke diagnosis and prognosis (152, 156). A refined gene expression signature would allow a readily available clinical evaluation by blood test, especially when brain imaging facilities are limited (153). Unraveling the differential expression of transcriptomic profile in the whole blood as well as the isolated immune cell populations would not only help to understand the underlying mechanisms during the stroke pathology, but also aid in the development of novel treatments for stroke. Due to RIC's early potential in reducing brain ischemic infarct caused by severe AIS, evaluation of transcriptome in peripheral blood cells following RIC and its comparison with stroke-related changes in gene expression may provide key mechanistic insight into neuroprotection.

The first study of genome expression in human leukocytes following RIPreC was reported by Konstantinov et al. who found that conditioning stimulus achieved by transient forearm ischemia (3 cycles of 5 min I/R) in healthy individuals significantly downregulated the expression of proinflammatory genes in leukocytes (46). These suppressed genes are known to be responsible for the inflammatory responses, including genes involved in TLR4-signaling, proinflammatory cytokine release (TNF-α), leukocyte chemotaxis and extravasation (PI3KCA), leukocyte adhesion (e.g., integrins, ADAM 8,10, PECAM), and exocytosis and secretory granule release (SNAP-23) (46). In another study by the same research group in 2010, these alterations in human leukocyte gene expression showed strong correlation with functional responses of neutrophils, in particular a significant reduction in neutrophil adhesion and phagocytosis ability (40) (Figure 3).

RIPreC was also associated with a 3-fold reduction in synaptosome-associated protein (SNAP-23) in leukocytes, a protein known to mediate exocytosis in mast cells and neutrophils. Lack of SNAP-23 prevents the formation of ternary complex with other SNARE proteins and therefore inhibits the fusion of granules in these cells (46). It is well-established that neutrophils mainly contribute to the inflammatory responses through secretion of specific cytoplasmic granules containing cytotoxic species and proteolytic enzymes; therefore, RIPreC-induced downregulation of SNAP-23 gene may partially explain the mechanisms that underlie the protection (46, 157). In addition, decreased levels of platelet endothelial cell adhesion molecule (PECAM1 or CD31) gene expression after RIPreC may be responsible for the observed reduction in the chemotactic ability of neutrophils (46, 157). PECAM1 is known to stabilize and preserve the integrity of BBB, and it is normally expressed on endothelial cells, platelets, neutrophils, monocytes and specific members of leukocytes. However, in neuroinflammation, PECAM1 mediates paracellular diapedesis across the vascular wall and its blockage abolishes the leukocyte migration (158). Therefore, lower expression of PECAM1 mRNA in RIPreC group compared to the controls may reduce the neutrophil transmigration in the brain (46).

RIPreC also suppresses the expression of the CCR2 gene that encodes for C-C chemokine receptor type 2, an essential protein needed for monocyte migration, infiltration and macrophage trafficking. This result is aligned with a significant reduction in the number of tightly adherent leukocytes and reduced leukocyte accumulation at the inflammatory sites in CCR2-deficient mice, suggesting that CCR2 modulation by RIC may reduce leukocyte adhesion (159). Taken together, RIC modulatory effects on immune responsive cells results in attenuation of inflammatory responses. This modulation includes reduction of excessive release of proinflammatory mediators during AIS, and enhancing the release of anti-inflammatory cytokines such as IL-6 and IL-10 (46).

## RIC: Role of EVs in Transferring the Protective Signal

Extracellular vesicles (EVs) are submicron-sized membrane-derived particles that are generated from different cell types under physiological and pathological conditions (160). Their contents include lipids, proteins, and genetic materials (i.e., microRNAs and circRNAs). EVs function *via* transferring their cargo, especially miRNAs, to neighboring target cells, or can act over long distances as an intercellular messenger (160). Fundamental biological processes in the target cells (e.g., proliferation, apoptosis, survival, and differentiation) can be modulated by EVs (160). EVs are classified into three main groups based on size distribution, chemical composition, and route of biogenesis: exosomes (30–150 nm), microvesicles or microparticles (MVs or MPs, 150–1,000 nm), and apoptotic bodies (500–5,000 nm) (160). However, since there is no strict size distribution for these sub types, and because different physiological or pathophysiological situations may affect their size and surface protein expression, it is recommended by the International Society for Extracellular Vesicles (ISEV) to use the general term “Extracellular Vesicles” while referring to the three subsets (161). EVs inherit their composition and physicochemical properties from their parent cells (162). Other than general EV markers, like tetraspanins CD9, CD63, and CD81, EVs carry signature markers of their cells of origin (e.g., common surface markers in humans are CD146+ for endothelial-derived EVs, CD41+ for platelet-EVs, CD45+ for leukocyte-EVs, and CD235+ for erythrocyte-EVs) (163). The ubiquitous nature and abundant presence of EVs in most body fluids and their ability to reflect cellular and molecular alterations under pathological states qualify them as promising and powerful tool in biomarker studies (163). For instance, platelet-derived microvesicles (PMVs) are known to play a key role in the pathogenesis of acute atherothrombotic events, such as thrombosis, recurrent ischemia, stroke, and vascular inflammation (164). Notably, there is a correlation between an increase in microvesicles released from platelets and endothelial barrier dysfunction (164, 165). Under normal physiological conditions, the majority of the circulating EV population is derived from circulating platelets and platelet precursors in the bone marrow (163–165). However, EV number, origin, and composition can change in pathology (164).

PMVs are important mediators of vascular homeostasis, inflammation, and angiogenesis (165). Accordingly, PMVs can contribute to the vascular homeostasis by maintaining the balance between their procoagulant and anticoagulant properties, depending on the composition of their surface markers or molecular contents. While the expression of phosphatidylserine (PS) and tissue factor (TF) on these vesicles can trigger the activation of coagulation cascades (166), the presence of glycoprotein 1b and annexin V is necessary for activation of protein C and its co-factor protein S, which are best characterized for their roles in anticoagulation pathways (167). PMVs can also play immunomodulatory role in modulating inflammation (165). PMVs exert pro-inflammatory actions mainly *via* provoking monocytes and neutrophils, thereby inducing them to release inflammatory mediators, including IL-1β, TNF-α, MCP-1, and MMP-9 (168). Notably, PMVs can boost the immune response by promoting leukocyte-endothelial interactions (169), *via* PMV uptake by activated neutrophils (polymorphonuclear cells, PMNs) and endothelial cells (ECs). Activation of neutrophils and ECs by PMV uptake was confirmed by an increased surface expression of CD11b and adhesion molecules (ICAM-1 and P-selectin), respectively (169).

Endothelial-derived microvesicles (EMVs) are also important players during various aspects of inflammation (170). They are believed to be secreted from activated ECs as an early response to any alterations in vascular homeostasis. In particular, EMVs contribute to fundamental processes affecting vascular endothelial cell fate, such as apoptosis, cell survival and proliferation, and homeostasis (170). A recent study investigated the content and vascular effects of endothelial-derived microvesicles (EMVs) during inflammation (171). It was shown that the protein levels of c-Src kinase inside the isolated EMVs from mouse was elevated (171) and led to dissociation of endothelial adherens junctions and increased vascular permeability (171). Additionally, src kinase hyperactivity caused higher adhesion and interaction between neutrophils and ECs, as was shown by elevated expression of adhesion molecules (ICAM-1 and VCAM-1) and integrins (CD11b) on the endothelium and neutrophils, respectively (171). In a rat model of focal brain injury and CNS inflammation (induced by IL-1β microinjection into the striatal region), Couch and colleagues demonstrated that the number of circulating CD31-positive EVs (i.e., endothelial cell origin) significantly increased in the acute phase of brain injury compared to age-matched controls (172). Proteomic analysis revealed that circulating EVs in the bloodstream after stroke contain upregulated proinflammatory proteins and can activate peripheral immune cells to induce an inflammatory response (172).

Numerous studies have been conducted to identify the conveyor of RIC protective signals from the remote site to the target organ. EVs may be a potential carrier of this signal (40, 173–177). EVs can transmit cargo (e.g., lipids, proteins, and nucleic acids) from the donor cells to nearby or far-away target cells to modify biological processes in them (178). In this regard, Shan et al. investigated whether transfusion of isolated PMVs from RIPreC-treated rats (donor) to rats who underwent transient MCAO (recipient) can confer protection (179). Their findings revealed a significant increase in PMV (CD41+ and annexin V+) levels in the PMV-treated recipient mice compared to control mice, with a resultant reduced infarct size and better neurologic outcome, indicating that PMVs may be a carrier of the RIPreC protective signal (179). Likewise, Li et al. reported neuroprotection conferred by RIPreC (three cycles of 10-min occlusion/reopening of mouse hindlimb) against permanent MCAO in mice was associated with increased levels of exosomes (<100 nm in diameter) in plasma (180). Interestingly, the transfer of purified exosomes from RIPreC-treated mice (donor) to non-treated stroked mice (recipient) reduced infarct volume and improved neurologic outcome in the recipient mice, indicating RIPreC protective signal may be conveyed through exosomes. Furthermore, RIPreC treatment upregulated the HIF-1α in the purified exosomes compared to the control group (180). Notably, sublethal hypoxic or ischemic conditioning also upregulates transcription factor HIF-1α, which in turn translocates into the nucleus and dimerizes with HIF-1β (181). After dimerization, HIF-1α binds to the hypoxia response elements on specific target genes, such as VEGF and erythropoietin (EPO), thereby counteracts the cell apoptosis. The neuroprotective properties of VEGF and EPO in ischemic brain have been linked to their ability to induce angiogenesis and neurogenesis, respectively (182, 183).

Similarly, whether RIC cardioprotection is transferable from the RIC-treated subject's plasma to naïve untreated subjects, and if this is mediated by circulating EVs, has been examined in both rodents and humans (184–186). Notably, these studies suggest RIC increased the release of EVs from the heart, RIC-induced protective signal is conveyed in part to the target organ *via* EVs, and this protection is transferable intra and across species. Using an *ex-vivo* langendorff-perfused rat heart method, Giricz et al. assigned the isolated hearts to two “donor” and “recipient” groups (173). A group of donor hearts were preconditioned *via* 3 alternate cycles of 5 min ischemia/5 min reperfusion prior to 30 min of global ischemia. Western blots against the EV marker (HSP60) revealed higher EV levels in coronary perfusates collected from preconditioned donor hearts compared to untreated control hearts, (173) as well as a smaller infarct, suggesting an EV-mediated transmission of RIC protective effects. Infarct size was significantly decreased in naïve hearts that received the coronary perfusate from the preconditioned donors, while no reduction of infarct size was noted in the hearts recipient of EV-depleted coronary perfusate (173). Likewise, RIPreC-induced cardioprotection has been associated with increased EV concentration and differential expression of specific microRNAs in plasma from patients who underwent coronary bypass surgery (187). Isolated EVs from RIC-treated patients added to cultured rat cardiomyoblasts *in vitro* conferred the same protection against hypoxia, indicating RIC protection is mediated *via* circulating EVs, and it is transferable across species (187). These studies also confirmed the presence of a well-recognized endothelial surface marker (i.e., CD146) on the isolated EVs, suggesting endothelial cells as a likely cellular source of RIC-induced EV release (187).

## Role of Micrornas in Transferring the RIC Protective Signals

MicroRNAs (miRNAs or miRs) are a key regulator of many fundamental cellular and molecular processes, such as cell growth, differentiation and apoptosis (188). MiRNAs are small single-stranded non-coding nucleic acids (~22 nucleotides long), which function through base-pairing with a complementary region in mRNA transcript and repress their translation into functional proteins (188).

The emerging role of miRNAs in stroke pathogenesis has recently been the focus of investigations in this field (189). MiRNA and target mRNA expression levels can change rapidly after the cerebral ischemia (190–193). Besides, numerous studies demonstrated that RIC alters the miRNA profile and thereby the expression and translation of genes and proteins. This again can reprogram the transcriptional response to the ischemic event (176, 194–196). Therefore, identifying the miRNAs and miRNA targets involved in stroke pathophysiology appears to be a promising candidate as either diagnosis or therapeutic tool.

Evidence from animal studies of cerebral ischemia suggests that miRNA (non-coding genes) genes may have a higher sensitivity to preconditioning (PC) stimulus than protein-coding mRNAs (191, 197, 198); since differential expression was observed in more than 20% of miRNAs, while <5% of coding mRNAs changed following PC application (197, 198). MiRNA profiling analysis by Dharap et al., in the cerebral cortex of preconditioned mice after a 10 min MCA occlusion reported a rapid change in the expression of 51 miRNAs following PC. Bioinformatics and pathway analysis suggested MAP Kinase and mTOR signaling are the main downstream signaling pathways of up-regulated miRNAs (26 out of 51), and Wnt and GnRH signaling pathways are the main targets of down-regulated miRNAs (25 out of 51) (197). Of these 51 differentially expressed miRNAs, the most up-regulated and down-regulated miRNAs 24h after PC were miR-21 (13-fold) and miR-466c (27-fold), respectively. Notably, miR-21 is anti-apoptotic factor that attenuates the expression of certain pro-apoptotic genes, including programmed cell death 4 (PDCD4), phosphatase and tensin homolog (PTEN), tropomyosin1 in neurons (199–203).

miRNAs play an important role in apoptotic signaling pathways through regulation of many pro-apoptotic genes (204–207). For a detailed review on apoptotic factors regulated by many different miRNAs, see Jang and Lee (204). For instance, Wu et al. demonstrated that upregulation of miR-21 (measured by rt-PCR Quantitative Kit) in human keloid fibroblasts were associated with a host of cellular events, which all led to the inhibition of cell apoptosis, including lower ROS, increased ratio of Bcl-2/BAX, decreased cytochrome C release into the cytosol, lower activity of caspase-3 and 9. All these events are critical components involved in the mitochondrial-mediated apoptotic pathway (206). In a rat embolic MCA occlusion model, Buller et al. demonstrated that elevated miR-21 level after focal ischemia attenuated the expression of Fas ligand (FasL) *via* complementary base-pairing with FasL transcript and blocking its translation into FasL protein ligands (208). Thus, miR-21 attenuates the neuronal cell apoptosis in the ischemic brain area by targeting critical cell death-inducing factors (200, 202, 203). FasL belongs to the TNF family, and its binding to one of the apoptosis signaling receptors FasR (apoptosis antigen-1, APO-1) initiates a cascade of events that leads to the activation of caspases and eventually causes neuronal cell death (209).

An important consideration is the potential use of miRNAs in maintaining the ionic balance in the ischemic region of the brain (176). During brain ischemia, there is a disruption in sodium and calcium balance due to downregulation of necessary ion channels and transporters by regulatory effects of some miRNAs (210, 211). To this end, blocking the expression of these miRNAs can be a therapeutic strategy to interfere with their detrimental behaviour (212). Anti-miRNAs evolution represents an efficient approach to inhibit and alter the action of miRNAs. Interestingly, in a rat model of transient cerebral ischemia, miR-103-1 was shown to downregulate the expression of Na/Ca exchanger (NCX1), a plasma membrane transporter which regulates the ionic homeostasis in ischemic brain. Notably, anti-miR-103-1 could significantly upregulate the expression of NCX1 mRNA and proteins levels in the brain cortex and striatum of ischemic rats, inducing a strong neuroprotective effect (~60% reduction in infarct volume) (211).

Aside from the regulatory role of miRNAs in the underlying mechanisms of conditioning-induced neuroprotection, miRNAs have also shown to be involved in the cardioprotective effects of conditioning (184). For example, Lassen et al. demonstrated that beneficial effects of RIC are delivered through EVs and their miRNA content. The transferability of EV-mediated RIC cardioprotection from the RIC-treated patients to *in vitro* cultured murine myoblasts was also demonstrated (184). In this study, the three miRNAs were most upregulated in association with cardioprotection after the RIC treatment were miR-144-3p, miR-451a, and miR-16-5p. These miRNAs demonstrated a two-fold upregulation, and each was linked to fibroblast growth factor 2 (FGF2) mRNA. Pathway analysis and gene ontology analyses suggested that all three differentially expressed miRNAs are associated with the mTOR signaling pathway and mediate the protein turnover, stress response, and apoptosis (184). Additionally, IR injury in the mouse myocardium reduces miR-144 expression levels, and this was reversed in mice receiving either RIPreC or systemic injection of miR-144 into the tail vein 30 min prior to global ischemia. These manipulations resulted in a marked reduction in infarct size and improved functional recovery of the heart. However, these beneficial effects were abolished after systemic injection of antagomir-144 (specific antisense oligonucleotide against miR-144), indicating the significance of miR-144 in RIPreC -induced cardioprotection. Moreover, miR-144 elevation led to downregulation of mTOR. Of note, mTOR signaling is an inhibitor of autophagy, which is a vital regulator of cell survival and a natural homeostatic mechanism of cell to remove the unnecessary or damaged components. Improved functional recovery after RIPreC may involve suppression of mTOR signaling and improved cardiomyocyte survival through increased autophagy (213).

Characterization and identification of EVs provides a “snapshot” of the environment of their origin cell at any given time. Additionally, they have a ubiquitous nature with high abundance in most body fluids. These features, along with their capacity as a vehicle for intercellular communications, position them as an ideal diagnostic and/or therapeutic target in many pathological states, including AIS. Moreover, these natural lipid mediators can be modulated for the delivery of specific agents or drugs to the target cells or organs, exhibiting superior properties relative to synthetic nanoparticles, including natural targeting ability, biocompatibility and safety. Thus, by identifying key EV-based mechanisms of RIC, new avenues of therapy to improve outcome after AIS can be developed.

## Author Contributions

SA-H researched, wrote, and designed figures for the manuscript. IW and GJ designed, co-wrote, and edited the manuscript with SA-H. All authors contributed to the article and approved the submitted version.

## Funding

Funding for this project was provided by the Canadian Institutes for Health Research (IW), Heart and Stroke Foundation of Canada (IW), and the David Lawson Graduate Scholarship (SA-H, Faculty of Medicine and Dentistry, University of Alberta).

## Conflict of Interest

The authors declare that the research was conducted in the absence of any commercial or financial relationships that could be construed as a potential conflict of interest.

## Publisher's Note

All claims expressed in this article are solely those of the authors and do not necessarily represent those of their affiliated organizations, or those of the publisher, the editors and the reviewers. Any product that may be evaluated in this article, or claim that may be made by its manufacturer, is not guaranteed or endorsed by the publisher.
